# openSNP–A Crowdsourced Web Resource for Personal Genomics

**DOI:** 10.1371/journal.pone.0089204

**Published:** 2014-03-19

**Authors:** Bastian Greshake, Philipp E. Bayer, Helge Rausch, Julia Reda

**Affiliations:** 1 Molecular Ecology Group, Biodiversity & Climate Research Centre, Frankfurt am Main, Germany; 2 Department for Applied Bioinformatics, Institute for Cell Biology and Neuroscience, Goethe University, Frankfurt am Main, Germany; 3 School of Land, Crop, and Food Sciences, University of Queensland, Brisbane, Australia; 4 Australian Centre for Plant Functional Genomics, School of Agriculture and Food Sciences, University of Queensland, Brisbane, Australia; 5 Hochschule für Technik und Wirtschaft, Berlin, Germany; 6 Johannes Gutenberg University, Mainz, Germany; Vanderbilt University, United States of America

## Abstract

Genome-Wide Association Studies are widely used to correlate phenotypic traits with genetic variants. These studies usually compare the genetic variation between two groups to single out certain Single Nucleotide Polymorphisms (SNPs) that are linked to a phenotypic variation in one of the groups. However, it is necessary to have a large enough sample size to find statistically significant correlations. Direct-To-Consumer (DTC) genetic testing can supply additional data: DTC-companies offer the analysis of a large amount of SNPs for an individual at low cost without the need to consult a physician or geneticist. Over 100,000 people have already been genotyped through Direct-To-Consumer genetic testing companies. However, this data is not public for a variety of reasons and thus cannot be used in research. It seems reasonable to create a central open data repository for such data. Here we present the web platform openSNP, an open database which allows participants of Direct-To-Consumer genetic testing to publish their genetic data at no cost along with phenotypic information. Through this crowdsourced effort of collecting genetic and phenotypic information, openSNP has become a resource for a wide area of studies, including Genome-Wide Association Studies. openSNP is hosted at http://www.opensnp.org, and the code is released under MIT-license at http://github.com/gedankenstuecke/snpr.

## Introduction

The availability of new DNA sequencing techniques has shifted the focus of biological data acquisition towards new biomedical applications. Many illnesses - for example Alzheimer's [1], Parkinson's [2] or different types of cancers [3], [4] - are at least partially heritable, so the genome of patients can be used for diagnostic purposes. Using the genetic information of patients for diagnostics is made possible through the sharp decrease in costs for analysing genetic information [5].

If genetic information on more than one individual is known, the analysis of allele frequencies of Single Nucleotide Polymorphisms (SNPs) can be used to associate such SNPs with illnesses and other inheritable traits. Genome-Wide Association Studies (GWAS) make use of statistics to compare the allele frequencies in patients to the alleles in healthy controls. This enables GWAS to find SNPs which are significantly overrepresented in patients and associates those SNPs with a trait or illness. While the method does not allow inference of causal differences but merely identifies correlations, it can serve as a valuable tool for the unbiased discovery of candidate loci, which then can be checked up in functional follow-up studies [6], leading to a deeper understanding of diseases and thus potentially to new drug targets. The first GWAS was published in 2005 and compared age-related macular degeneration in contrast to a healthy control group [7]. Since the beginning, the number of participants in such studies has been rising. To date, over 1200 GWAS have been performed [8] and over 5000 SNPs have been linked to different illnesses and traits [9].

GWAS are not only performed inside the traditional scientific community. Since 2006, companies like 23andMe, deCODEme or FamilyTreeDNA have been offering Direct-To-Consumer (DTC) genetic testing. These companies use DNA microarrays to screen for around 0.5 to 1 million SNPs spread over the human genome. In return, customers receive an analysis of the results, as well as a raw file that includes the customer's individual genotypes. In 2011, 23andMe alone had over 100,000 customers [10]. The company realizes the potential of performing GWAS with this amount of data by using surveys to ask their customers about traits and illnesses. With the consent of the customer, the data is used for association studies. 23andMe has published several studies in which known findings are replicated together with new associations for disorders like Parkinson's Disease [11], [12]. So far, over 30,000 23andMe-customers have participated in 23andMe's association studies, which proves that this data source has a lot of potential for other researchers.

The generation of biomedical data by private companies raises concerns about privacy [13], liability and consent [14]. Nevertheless, in some instances individual customers are willingly sharing their data. Most do so by uploading their data to their personal website or to open software repositories like *GitHub*. This data is scattered and unorganized, making it hard to use in studies. While projects like SNPedia try to keep track of all the publicly available genotyping files [15], they usually do not provide the information necessary to perform GWAS, as the phenotypic information is often not attached to the genetic information. Projects that attach the phenotype to the genetic information, like the *Personal Genome Project* [16], still do not allow for an easy re-use of the data, as they currently lack an application programming interface (API) or other methods by which researchers could download the data. Additionally, not every customer of DTC genetic testing can participate in the *Personal Genome Project*, as their consent forms only allow residents of the United States to apply.

Crowdsourcing, giving a task into the hands of a potentially large number of – mainly intrinsically motivated – people, has become a widely used practice in the internet age and is getting adopted in the realm of science as well. One of the main benefits of crowdsourcing is that small contributions to a project pile up to create a larger work, which would have been virtually impossible to create otherwise. This approach especially benefits scientists who might not have enough funding or time to create data, or in cases where the amounts of data are too large to be analyzed by researchers alone. Galaxy Zoo and FoldIt [17], [18] are two of the best known examples. Galaxy Zoo enables amateur astronomers to walk through telescopic images to categorize the shown objects at a rate which could not have been matched by the efforts of professional astronomers. Similarly, crowdsourcing can not only be applied to analyzing data, but also to collecting data. This approach has been shown to work when it comes to tracking bird migration [19]. With the advent of DTC genetic testing and the internet, a similar approach can now be applied to human genetics.

There have been studies investigating how likely customers of such companies are to share their data. [20] investigated the likelihood of 2,024 individuals to share their test results with their health-care providers and found that 26.5% (540 individuals) did share their results with their physician or health-care provider. Those that shared were older, had a higher income and were less concerned about testing or the privacy implications of sharing their data compared to customers that didn't share their data.

Other studies have shown that DTC customers see themselves as being well-informed. Interviews with early adopters have shown that these customers are better informed and more skeptical about the capabilities of genotyping than expected [21]. However, in another study of early adopters, 32% of customers had misperceptions about personal genomic testing [22]. Of these participants, 92% intended to share their results with physicians in order to receive medical recommendations. In both studies, participants generally chose this technology to be better informed about genetic risks and to satisfy their own curiosity.

Here, we present openSNP, an online platform which enables DTC customers to share genotypic and phenoytypic information, as well as receive additional information on their genotypes. The genotypes are made available to researchers via the open Creative Commons Zero license.

## Results

### Sharing genotypic information

We created the openSNP project (http://opensnp.org) as an open, crowdsourced online platform for DTC customers interested in sharing their raw data and for researchers interested in performing GWAS or other types of analysis with the data. Customers of DTC testing are encouraged to share their genotyping results along with their phenotypic traits to enable easy access for researchers. Users of openSNP can create a personal profile, discuss SNPs and phenotypes on the platform using a simple commenting system, or send each other private messages.

People interested in using the data of openSNP can download complete dumps of the genotypic and phenotypic information or use query API endpoints utilizing JavaScript Object Notation (JSON) objects or the Distributed Annotation System (DAS) [23].

**Figure 1:**
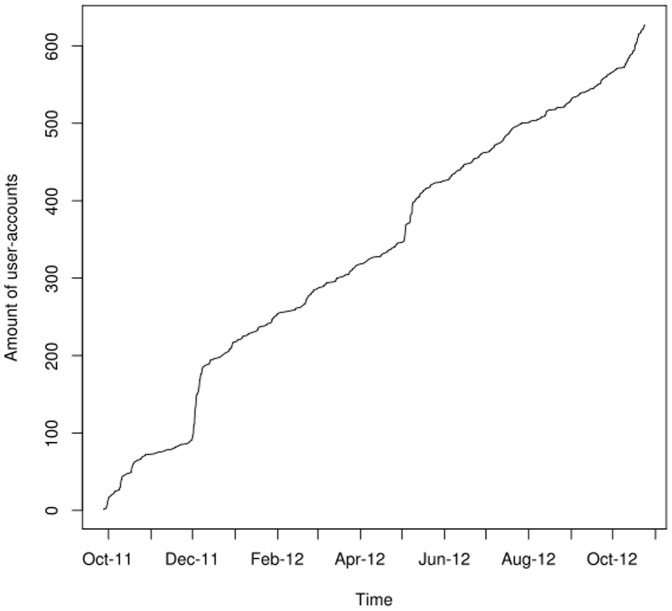
Growth of openSNP-user-accounts. The increase in numbers for users from 27.09.2011 to 27.10.2012 is shown.

**Figure 2:**
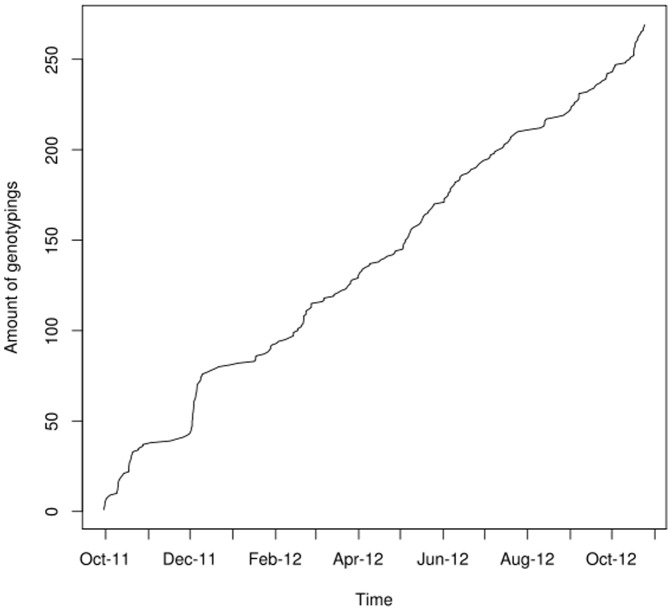
Growth of available genotypings. The increase in numbers for genotyping-files from 27.09.2011 to 27.10.2012 is shown.

Currently users can upload their genotyping results from the companies *23andMe*, *deCODEme* and *FamilyTreeDNA* via a web interface to the openSNP project. There is experimental support for uploading exomes in the VCF format [24], as *23andMe* recently started exome sequencing for its customers. Due to space constraints on the database level, openSNP currently only displays the SNPs of the exome data sets on the website but the whole VCF files can be downloaded. The uploaded data is published under the Creative Commons Zero license, which – in accordance with the Panton Principles [25] – allows a complete re-use of the data without any constraints. Between the launch of openSNP on 09/27/2011 and 10/27/2012, 633 people have signed up with openSNP, and 270 genetic datasets have been made available. As of 10/27/2012, the openSNP database lists 215,546,685 genotypes which are distributed over 2,140,643 unique SNPs. Figures 1 and 2 depict the increase in users and genotyping files since September 2011.

### Crowdsourcing phenotypes

Users are able to create new phenotypes that are not yet listed by openSNP. The specification of these phenotypes is open and not limited to pre-defined categories. To reduce the amount of manual data curation, openSNP tries to harmonize the expression and spelling of the same phenotype or variation. We implemented an autocompletion feature, which helps users reuse already entered phenotypes. Users are encouraged to list as many phenotypes as possible through a simple achievement system, rewarding users that upload their data and enter phenotypic information with badges that are shown on their profile pages.

**Figure 3:**
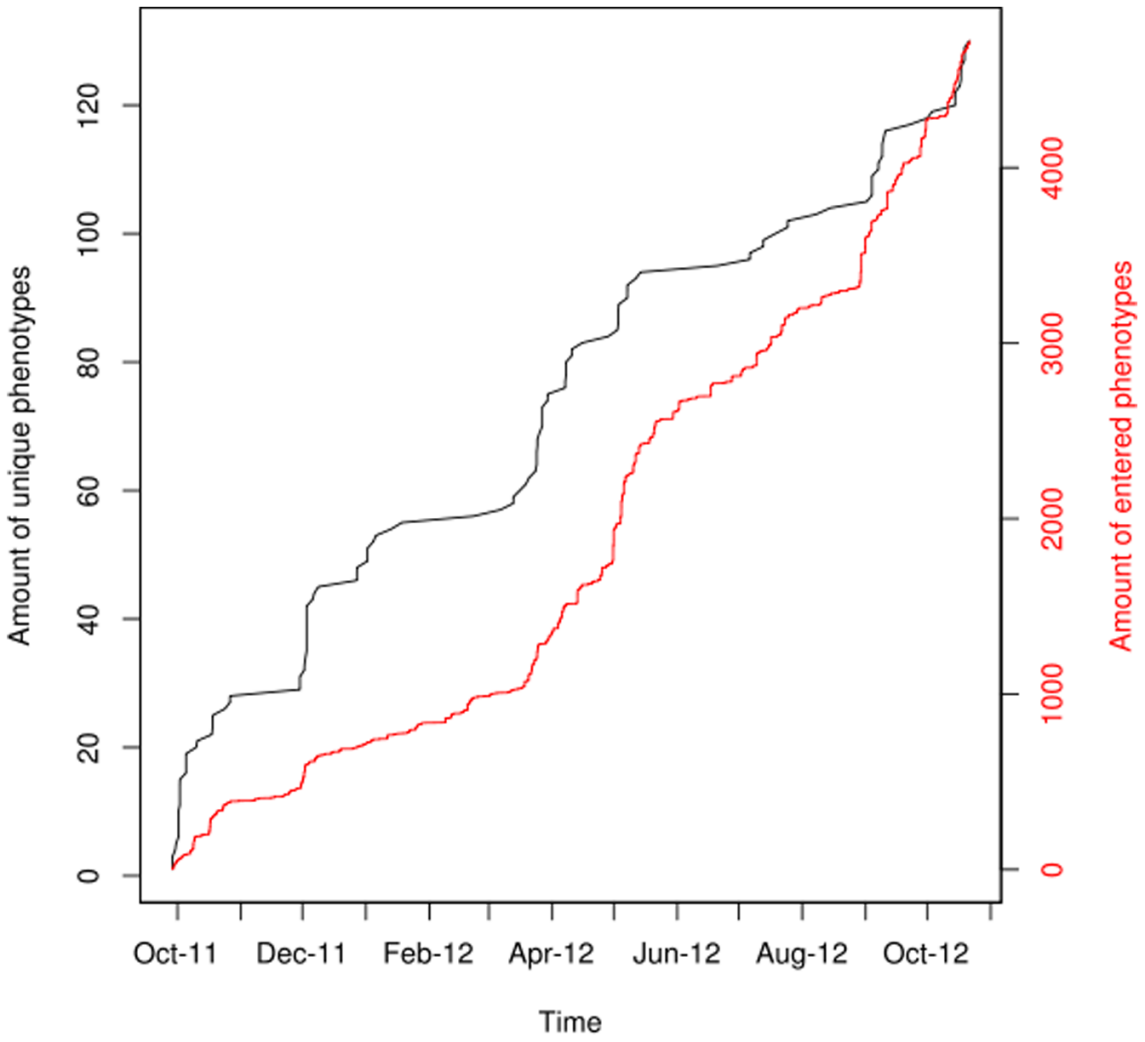
Development of unique phenotypes and phenotypic information over time. The x-axis shows the time-frame from start of the project until October 2012, the left y-axis shows how many unique phenotypes have been entered, and the right y-axis shows the amount of phenotypes users entered.

In the same timeframe mentioned above, all users combined have entered a total of 4743 variations on 130 different phenotypes with those variations being the different values on a given trait or phenotype. The mean number of users that have entered their variations for a single phenotype is 36.48. The distribution of how many users have entered their data per phenotype, compared to the amount of unique phenotypes, can be seen in Figure 3. The phenotype provided by the most users is “eye color”, for which 207 users entered their phenotype (retrieved 10/27/2012).

### Connection to external services

**Figure 4:**
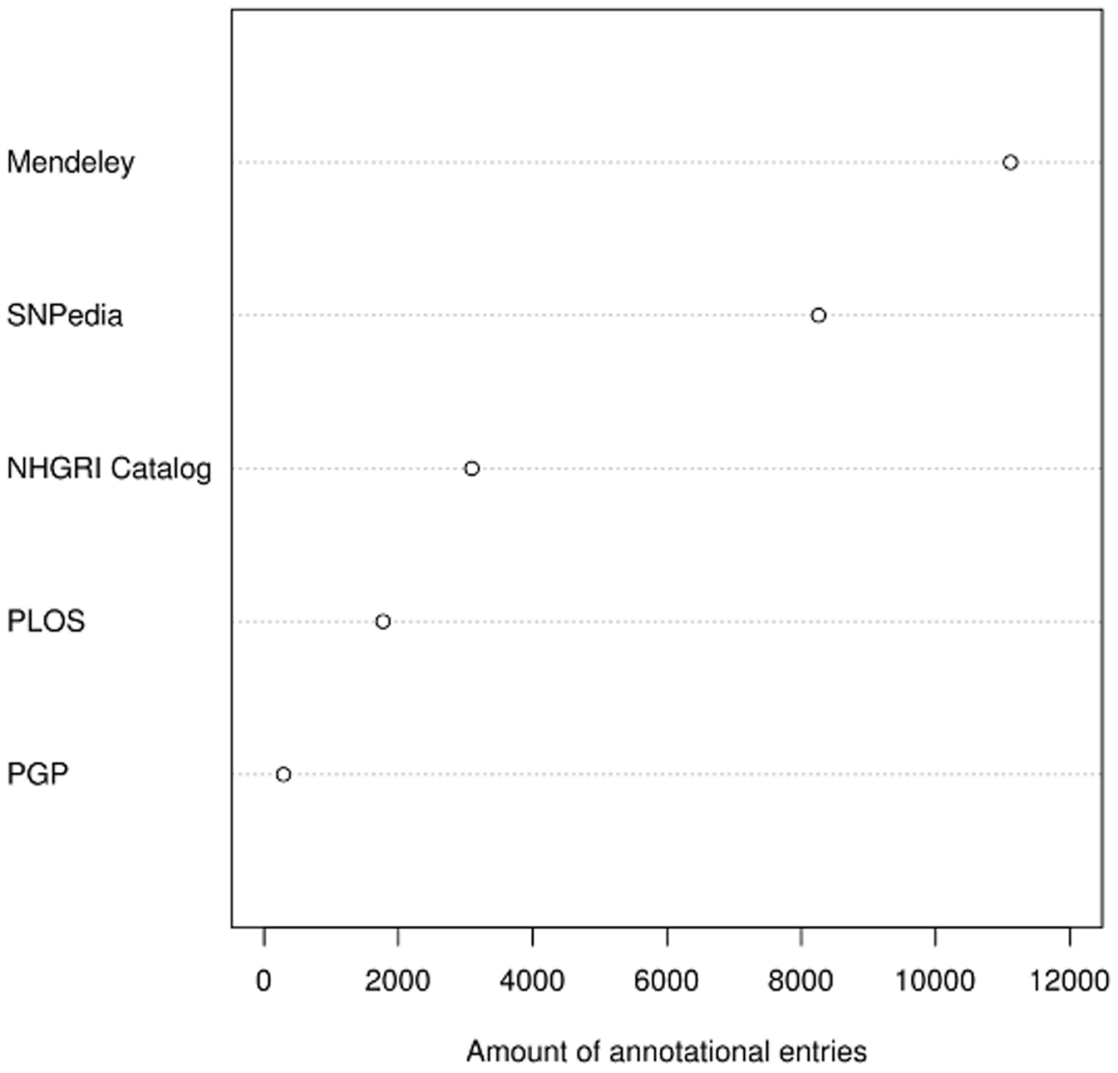
Distribution of annotation-sources at openSNP. Currently, SNP-annotations from SNPedia, PLOS, Mendeley, the *GET Evidence System* and the *NHGRI GWAS Catalog* are being collected.

**Figure 5:**
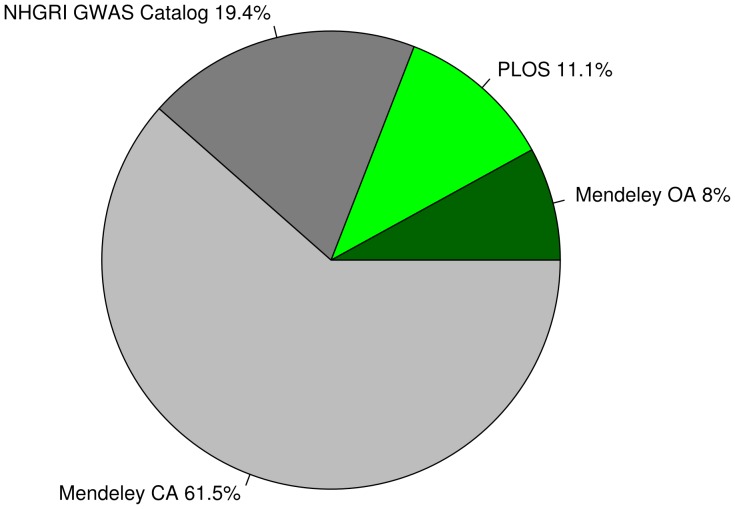
Ratio of open access Publications. Green pieces are open access. The *NHGRI GWAS Catalog* doesn't give information about the open access status.

In order to provide users with relevant information on their respective genotypes, openSNP scans databases of the scientific literature for specific SNPs. A total number of 21,134 documents relevant to the SNPs listed in openSNP could be found in the publication and annotation databases of Mendeley, the Public Library of Science, in the *GET Evidence System* [16] and the *NHGRI GWAS Catalog* [9] and in the crowdsourced SNPedia (Figure 4). Of the primary literature listed on Mendeley, the *NHGRI GWAS Catalog* & the Public Library of Science, about 20% are released in open access journals and can be accessed free of charge (Figure 5), although probably not all publications on Mendeley are correctly flagged and the *NHGRI GWAS Catalog* does not give details on whether a publication is open access or not. So the total number of open access publications might be higher.

For usability reasons, SNPs are ranked by the amount of information gathered through the external services. The external services themselves are ranked by how easily non-scientists can understand information from these sources and how available this information is to the public. The SNPedia entries are given the highest impact, as those are already manually curated and summarized in plain English, followed by open access publications out of the Public Library of Science and the curated databases of the *GET Evidence System* and the *NHGRI GWAS Catalog*. Lowest values are given to the Mendeley results, as the publications listed there are for the most part not freely available without subscriptions or one-time payments. An entry on SNPedia is valued 2.5 times as high as a PLOS publication or entries in *GET* or the *GWAS Catalog* and 5 times as high as a Mendeley entry.

Users are also able to link their Fitbit[26] accounts to their user-accounts. Fitbit is a commercial service which lets its customers track their BMI, movement and sleep data. This data can be linked to openSNP to give interested researchers an automatically maintained dataset of body and sleep developments over time.

### Data access

openSNP offers complete access to the data uploaded by users. Anyone can download single genotyping files for specific users, get archives of multiple genotyping files grouped by phenotypic variation, or access a single download that includes all genotyping files and all phenotypic variation in a comma-separated table. For privacy reasons, openSNP does not log any IPs. The genetic data is also accessible through the Distributed Annotation System [23], [27], which offers all data for specific chromosomes and specific positions on single chromosomes. An example of how the DAS can be used is implemented on openSNP, where users' genotypes are visualized inside a genome browser. All chromosomal positions are based on the human reference genome NCBI37, as this is the standard reference used by DTC providers right now.

The data is additionally available over a JSON API which allows users to directly access data in the JSON format. The methods allow users to programmatically look for the genotypes and annotations at a given SNP as well as for phenotypes for a given user and phenotypic variation for a given phenotype.

## Discussion

Here, we present openSNP, a crowdsourced resource that enables customers of DTC testing companies to share their genotypings with researchers and receive new annotations for their genetic variants. Through a of number of active users already present on openSNP, we have shown that at least some customers of DTC companies are willing to share their data at no cost to researchers around the world and are willing to annotate their data with phenotypes.

### Comparing openSNP to other crowdsourcing platforms

Projects similar to openSNP are the SNPedia, the Personal Genome Project and PatientsLikeMe.com (see table 1 for an overview). The focus of the SNPedia is the aggregation and summary of primary scientific literature on SNPs. The project uses a Wiki to store and display the data collected by volunteers contributing to the project. The data is mainly organized by the unique Rs-ID, as given by dbSNP. If Rs-IDs are missing, the identifiers given by the DTC testing companies may be used, similar to the way openSNP stores the data. For individual SNPs, pages may list scientific literature and summaries on the found impact can be given. As those pages are largely created manually and not automatically through database access, these summaries may not be complete. openSNP utilizes the SNPedia by crawling their data for SNPs, the summary of the impact and the magnitude a SNP has. While they offer a page listing download-URLs, the SNPedia does not offer any uploading capabilities for genetic data and has no APIs to easily access SNPs or data subsets in the different data sets. Similarly, there is no way for users of SNPedia to share their phenotypes in a machine readable format.

**Table 1:** Comparison of crowdsourced genetics platforms.

Name	Provides	Provides	Open	API	IRB approval	License
	annotation	data	enrollment			
SNPedia	(x*)	-	N/A	x	N/A	CC-NC-SA 3.0
PGP	x	x	-	-	x	CC-BY 1.0, CC0
PatientsLikeMe	-	x	x	-	x	Closed, CC-BY-SA 3.0
openSNP	x	x	x	x	-	CC-BY 3.0

N/A  =  Not Applicable, x  =  Present, -  =  Absent *SNPedia only provides an API to webpages of individual SNPs, not access to genetic data of individuals.

The Personal Genome Project (PGP) has its focus on collecting and hosting genetic as well as phenotypic data. Unlike openSNP, they do not offer a completely open enrollment. For each participant of the PGP, eligibility has to be established and participants have to give IRB approved informed consent. This allows for an easier re-use of the data, but at the same time makes it impossible for many people to enroll (e.g. non-US citizens). Depending on the specific use one has for the data, the PGPs enrollment policy might be preferable to the open approach openSNP takes. While the PGP stores genotyping data as well as exome and genome data sets, it is currently impossible to access this data through an API, instead data has to be manually extracted from their database. The annotation database of the PGP is not aimed at delivering specific publications, but instead focuses more on specific traits. The annotation data stored by the PGP is incorporated into openSNP as well.

PatientsLikeMe is a community for patients with life-changing illnesses to track and share the development of their illness with other patients with similar illnesses [28]. This helps patients in gaining a better understanding of their illnesses – 72% of surveyed participants found the site “moderately” or “very helpful”, for example when it comes to starting a new medication (37% found the site helpful), or when it comes to changing the medication (27%). Some subsets of data stored in PatientsLikeMe are open to the public and have been shown to be useful for research, for example in Multiple Sclerosis [29]. Alternatively, access to the data they store can be licensed by researchers for a fee.

There are some projects that use gamification to let players work with crowdsourced scientific data. For example, FoldIt is a puzzle game that lets players fold protein structures in order to achieve optimal structures. Players of Foldit have been able to identify protein structures and were even able to improve the activity of existing protein structures [17]. Another example is Galaxy Zoo [18], which allows everyone to perform classification tasks for galaxies based on images collected by the Sloan Digital Sky Survey.

Unlike PatientsLikeMe, the PGP, or openSNP – which give the task of collecting the data into the hands of the crowd – Foldit and Galaxy Zoo limit themselves to analyzing data which was previously collected by scientists.

### Privacy, health implications and ethical considerations

Much of the criticism of DTC genetic testing focuses on the practice of delivering medical information without consulting a physician or genetic counselor to help patients/customers make sense of the information and to put the new knowledge to good use [30]–[32].

There is a variety of ethical and privacy implications when it comes to DTC genetic testing [14], [33], [34]. Nevertheless, studies show that DTC customers are willing to share their results given the right circumstances and personal benefits gained through the sharing, while being aware that sharing genetic data can lead to misuse of the data and consequences such as genetic discrimination [20].

As people are concerned about their privacy and fear that stakeholders like employers, insurance companies, governments or advertisers might misuse the information [35], policy makers start to react to those changes by having introduced laws like the *Genetic Information Non-Discrimination Act* (GINA) in the United States or the *Gendiagnostikgesetz* (GenDG) in Germany to minimize the impact of widely available genetic information. DTC genetic testing companies themselves also try to create online communities - like the 23andMe community forums - that help in educating their customers about the risks of releasing genetic data [36]. Neither GINA nor the GenDG offer complete protection from genetic discrimination, as certain areas, such as life insurances, are not covered by those laws.

openSNP openly addresses the problem of privacy implications that come with releasing genetic data twice, once during registration for openSNP and once during the upload of the DTC genetic testing results. Users have to confirm that they have read and understood the disclaimer about possible side-effects of publishing their data. Further versions of openSNP may include further consent processes.

For users of openSNP, the biggest potential problem is legal genetic discrimination, in fields not covered by laws such as GINA or GenDG, once their public data is re-identified. As the genetic information itself is highly personalized the anonymous sharing of genetic data is impossible. And while users can register pseudonymously, this should not be seen as ultimate protection against re-identification. A recent study once again showed that metadata, potentially attached to genetic profiles, such as date of birth, gender and postal code, can be be used to re-identify individuals on a name basis [37]. A similar approach utilized genetic markers on the Y chromosome along with genealogical databases and metadata such as age and state to infer surnames and from there on the individuals [38]. Thus users need to be aware of the potential of re-identification through providing metadata along with their genetic information and the genetic discrimination that could follow.

### GWAS and Open Data

Although prices of exome or even full genome sequencing are dropping rapidly, GWAS are still considerably cheaper. However, GWAS can only detect correlations of SNPs with those traits and do not allow inference on the cause for any correlation. Furthermore, for a statistically sound analysis, GWAS need a large enough sample size, which often is not easy to obtain. Either because generating the needed amount of data still is a cost factor or because it is hard to find enough participants for the case conditions, for example if rare diseases are to be studied. Nevertheless, GWAS are still frequently used and new associations are still being discovered [39]–[41].

One way of bringing down costs for GWAS even further is to make use of already available genotyping results and datasets. Data produced by DTC genetic testing companies is a promising source for such results, as those companies already have high numbers of customers who are willing to pay for the genotyping by themselves.

openSNP tries to enable and facilitate the re-use of this already generated data by offering a platform where customers of DTC genetic testing can publish their results into the public domain. Allowing interested parties to use the data for their own research allows scientists to perform studies without the need to generate genetic data sets on their own. Additionally the data can be used to enrich other data sets in order to overcome limited sample sizes, which is especially of interest for rare diseases.

In crowdsourcing the acquisition of genetic and phenotypic data, openSNP faces the same problems as any other open platform on the Internet, namely the need to trust users regarding the data they upload and enter on openSNP. Additionally, the quality of the data varies, especially in terms of accuracy on the phenotypic variation, with users entering data in different measurement systems. Another problem with user-entered data is the frequent switching between categorical and continuous phenotypes - for example, some users entered the specific value of their height, while other users entered their height according to a category like “150 cm to 160 cm”.

While we try to suggest similar entries to the users, there are some cases where users will not follow those suggestions, so duplicates or similar phenotypes or variations in traits may arise. There are three possible solutions to this problem: The first one would be to only allow a trusted subset of users to enter new phenotypes. The second one would be to make users enter all possible variations of a phenotype while creating a new phenotype, so that later users cannot add variations that have not been available from the start. The third one is to exclude users from the phenotype-creation process by allowing users to select their phenotypes from a pre-given set of possible variations.

In the first two cases it makes it harder for users to enter their data which raises the bar for participation, and the third case doesn't let users participate at all. We decided to keep data entry as easy as possible, at the cost of forcing users who want to perform GWAS with the data to perform additional quality control.

Another risk regarding data quality that should be kept in mind is a possible bias in data availability on openSNP: only a subset of people buy DTC genetic testing, from which an even smaller subset is willing to publish the results, which can potentially lead to skewed GWAS-results. 21 people, mainly from underrepresented demographics, have been offered free genotypings using funding provided by the Wikimedia Germany association in order to mitigate this bias.

Furthermore, it is impossible to verify whether users who have uploaded data are actually the sources of that data. This opens the venue to potentially malicious usage, as genotypings from strangers can be uploaded, as well as misinformation about phenotypes can be entered. The openSNP project has currently no means of verifying the validity of data uploaded by users. Of course, users can always delete their data or contact the team to delete stored data. Old backups of the database are deleted so that at any given time, there are only two backups. This means that deleted data disappears from the webpage immediately and will disappear after two months in the backend where it isn't accessible to the public.

With openSNP, we have built a platform that can be used by customers of DTC genetic testing to easily share their genetic and phenotypic data with a wide audience, as well as by scientists and interested citizens who are looking for datasets to freely use in their studies. Customers of DTC genetic testing also benefit from an easy access to primary literature on SNPs and genetic variations they carry. While there is not enough data uploaded to perform a statistically sound GWAS yet, this will be possible in the future, as user numbers continue to rise. By including the option of uploading exome data sets, the platform is already capable of adjusting for changes in the type of data generated by DTC genetic testing. Future improvements made on openSNP will address interoperability with other platforms and tools in Personal Genomics, amongst others: The standardization of phenotypes, the inclusion of further annotation sources and support for a wider range of data sets, including full genome data.

## Materials and Methods

### Ethics Statement

In line with the German regulations and the ethics approval system for biomedical studies [42] we contacted the ethics commission of the Goethe University Frankfurt am Main, Germany teaching hospital. Its director confirmed that this study does not fall within their remit.

### Technical implementation of the platform

The main platform is implemented using the web framework Ruby on Rails 3.2.13. Postgres 9.2 is used as the main database backend for Rails. The database stores genotyping results, users' phenotypic information, literature results from Mendeley and the Public Library of Science as well as summaries on SNPs which can be found in SNPedia. The literature database of Mendeley is queried using the REST API, which delivers results in JSON. The literature database of the Public Library of Science is queried using the respective REST API, which delivers results in an XML-format. Summaries on SNPs are provided by SNPedia, through querying the content via the MediaWiki API. The *NHGRI GWAS Catalog* and the *GET Evidence System* provide complete dumps in plain text formats. Those are regularly downloaded and parsed. SNPs that are described as ‘Insufficiently evaluated’ in the *GET Evidence System* are not stored. All databases are queried or parsed using the unique identifier of each SNP as the search term.

SNPs are catalogued by their unique identifier, which consists of a prefix (mostly *rs*, rarely *i*) and a unique number. This is a common format, which is employed by the NCBI dbSNP database [43] and is also widely used and easily parsed from different literature sources. Publications from the different databases as well as the users' genotypes are associated with individual SNPs by the Rs-ID. Allele and genotype frequencies are updated regularly, based on the data present in openSNP.

Processes with a longer runtime, such as parsing the genotyping results, creating archives of results which are to be mailed to users and queries to external resources are handled using the ruby gem Resque and the standalone key-value storage server Redis. Search features on the platform itself are implemented using Solr and the ruby gem Sunspot. Additionally, data can be requested from openSNP using the Distributed Annotation System. The required data is stored in a PostgreSQL database. Requested data is delivered in XML-format to facilitate parsing. Additionally, users can request data in the JSON-format, using a system not specified in any standard.

openSNP only serves as a platform for SNPs, so methods for the delivery of nucleotide sequences as described in the DAS-standard are not implemented. Currently, two methods are implemented: firstly *features*, which is used to deliver SNPs located on specific chromosomes or between specific nucleotide positions, based on the user's query. The second method is *sources*, which advertises all DAS sources for all genotypes present in openSNP.

**Figure 6:**
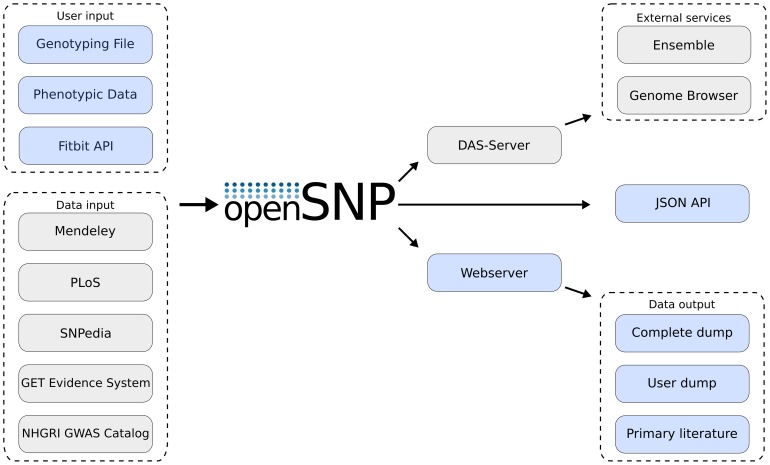
Flow of data inside openSNP. External databases and user-provided data are used as input. Output of data is done using the website, the *Distributed Annotation System* and a JSON-API.

A flowchart of all services incorporated in openSNP and of all the ways users can upload or access the data is given in Figure 6. The source code of openSNP is published under the MIT license and can be downloaded at http://github.com/gedankenstuecke/snpr. The genetical and phenotypical data is licensed under Creative Commons Zero.
